# Stratified phase II trial to establish the usefulness of the collagen gel droplet embedded culture-drug sensitivity test (CD-DST) for advanced gastric cancer

**DOI:** 10.1007/s10120-013-0320-4

**Published:** 2013-12-08

**Authors:** Hiroyuki Naitoh, Hiroshi Yamamoto, Satoshi Murata, Hisayuki Kobayashi, Katsunori Inoue, Tohru Tani

**Affiliations:** 1 Department of Surgery, Shiga University of Medical Science, Seta, Tsukiwa-cho, Otsu, Shiga 520-2192 Japan; 2 Department of Surgery, Hino Memorial Hospital, Shiga, Japan; 3 Bio-Medica Department, Kurabo Industries Ltd., Osaka, Japan; 4 Department of Clinical Laboratory, Medic Co., Ltd., Osaka, Japan

**Keywords:** Gastric cancer, Chemosensitivity test, Collagen gel droplet embedded culture-drug sensitivity test, CD-DST, S-1

## Abstract

**Background:**

We conducted a multicenter phase II trial to assess the suitability of three types of chemotherapy (docetaxel plus S-1, irinotecan plus S-1, or S-1 alone) for patients with advanced gastric cancer by means of the collagen gel droplet embedded culture-drug sensitivity test (CD-DST). To our knowledge, this is the first multicenter clinical trial that has employed CD-DST to choose anticancer agents for the treatment of advanced gastric cancer.

**Methods:**

Subjects (*n* = 64) were patients with advanced or recurrent gastric cancer. Patients were allocated to one of the treatment regimens on the basis of CD-DST results. Outcome of the patients was compared between the groups deemed chemosensitive or chemoresistant by the CD-DST.

**Results:**

Thirty-three patients showed high sensitivity (T/C ratio <60 %) to at least one type of anticancer agent (sensitive group), and 31 showed low sensitivity (T/C ratio ≥60 %) to all agents (resistant group). Specifically, the 1-year survival rate was significantly higher in the sensitive group (78.5 %; 95 % CI, 67.2–94.7 %) than in the resistant group (54.7 %; 95 % CI, 38.7–74.3 %; *P* = 0.019), whereas time to progression (TTP) was significantly longer in the sensitive group (59.8 %; 95 % CI, 48.2–81.7 %) than in the resistant group (30.0 %; 95 % CI 13.6–46.4 %; *P* = 0.023). Median survival time was also significantly longer in the sensitive group (15.5 months; 95 % CI, 12.8–18.2) than in the resistant group (12.5 months; 95 % CI, 10.2–14.9; *P* = 0.038).

**Conclusions:**

CD-DST predicts the outcome of patients undergoing chemotherapy for advanced gastric cancer, presumably through evaluating chemosensitivity.

## Introduction

The prognosis of patients with resectable gastric cancer has improved with the development of technologies that enable early diagnosis and progress in surgical technique and perioperative management. However, prognosis remains extremely poor for those with locally advanced or recurrent cases or those with distant metastasis. Several anticancer agents were recently introduced and have boosted the hope of better chemotherapy outcome. A regimen most commonly used globally both in the clinical practice and as a reference arm in phase III trials had been a combination of cisplatin (CDDP) and 5-fluorouracil (5-FU) [1–3]. Given the approval of several types of new drugs, however, we hypothesized that personalized therapy guided by adequate chemosensitivity testing could lead to superior outcome when compared with the empirical therapy. For this purpose, the collagen gel droplet embedded culture-drug sensitivity test (CD-DST), a new and sophisticated method, was expected to serve an important role. Thus, we organized the Shiga Clinical Study Group for Chemosensitivity Tests for Gastrointestinal Cancer, which comprises 20 participating institutions, and conducted a stratified phase II trial for the combination treatment of docetaxel (TXT) and S-1, irinotecan (CPT-11) and S-1, or S-1 alone in advanced gastric cancer guided by CD-DST. Participants were patients with advanced or recurrent gastric cancer who were treated with anticancer drugs according to CD-DST chemosensitivity results for TXT, CPT-11, and 5-FU.

## Materials and methods

### Objective

The objective of this study was to prove that CD-DST predicts survival of patients who undergo chemotherapy for advanced gastric cancer: that 1-year survival rate of patients who were determined as chemosensitive by CD-DST is significantly higher than that of those determined as chemoresistant.

### Eligibility

Patients with recurrent or advanced gastric cancer who suffer from either unresectable or residual disease were eligible. Availability of fresh tissue samples for CD-DST was also a prerequisite for enrollment. Gastrectomy in the current study was therefore performed either to palliate symptoms related to the primary lesion or as a reduction surgery. In addition, patients whose metastases were found during surgery were also eligible, provided the fresh samples could be harvested and transported immediately for CD-DST. Patients with unresectable primary lesion were eligible only when sufficient biopsy samples were available for chemosensitivity testing. Patients with recurrent disease were also eligible when fresh specimens of the recurrent cancer were available. Other inclusion criteria were as follows: age of 20–79 years; histologically proven gastric cancer; no previous chemotherapy and/or radiotherapy with the exception of adjuvant chemotherapy given after curative surgery. ECOG performance status score of 0–1; capable of oral ingestion; predicted survival of 3 months or more from the first day of chemotherapy; satisfactory function of bone marrow, heart, liver, and kidney; and ability to provide written consent. Between August 2007 and May 2009, 80 patients from 20 medical institutes in Shiga Prefecture, Japan, were enrolled in the study. The study was formally approved by the ethics committee of each participating institute and conducted in accordance with the principles of the Declaration of Helsinki. Written informed consent was obtained from all patients.

### CD-DST procedure

Immediately after surgical resection of the tumor in each patient, a viable portion of the tumor was identified and resected by a physician who was not involved in the surgery itself to avoid delaying the surgery. The resected tumor was immediately stored in culture medium at 4 °C, and CD-DST was started promptly on the same day as the surgery. A single operator (K.I.) performed all CD-DST assays at a laboratory in Shiga University of Medical Science to evaluate sensitivities to TXT, CPT-11 (SN-38), and 5-FU. The CD-DST procedure was performed similarly with the biopsy specimens when samples weighing 0.1–0.5 g in total were available.

CD-DST was carried out according to the method reported by Kobayashi et al. [4, 5], who invented the method using a human tumor cell primary culture system kit (Primaster^®^; Kurabo Industries Ltd., Osaka, Japan). Briefly, each sample was washed five times with 50 ml saline containing 1.0 mg/ml penicillin, 0.5 mg/ml kanamycin, and 2.5 μg/ml amphotericin B and treated afterwards with Dispersion Enzyme Cocktail EZ (Primaster^®^ reagent). Obtained cell suspension samples were inoculated into collagen gel-coated flasks (CG flasks, a Primaster^®^ device) and cultured overnight in pre-culture medium PCM-1 (Primaster^®^ content) at 37 °C in 5 % CO_2_. Next, the collagen gel was digested with 0.05 % EZ, and viable cancer cells were obtained. Type I collagen, 10× concentrated F-12 medium, and reconstitution buffer were mixed together in ice water with a ratio of 8:1:1 (Primaster^®^ content). The prepared cancer cell suspension was added to the collagen solution at a final density of 1 × 10^5^ cells/ml. Three drops of the collagen-cell mixture (30 μl/drop) were placed in each well of a 6-well plate on ice and allowed to gel at 37 °C in a CO_2_ incubator; the final concentration was about 3 × 10^3^ cells per collagen gel droplet. DF medium containing 10 % fetal bovine serum (FBS) was overlaid in each well 1 h later, and plates were incubated overnight in a CO_2_ incubator at 37 °C. The anticancer drugs were added at the following final concentrations and incubated for 24 h: 0.1 μg/ml TXT, 0.03 μg/ml CPT-11 (SN-38), and 1.0 μg/ml 5-FU. The concentration of each anticancer drug in the culture medium was determined so as to exhibit the same area under the curve value as observed in the serum during the first 24 h after the intravenous administration of the corresponding drug at the standard clinical dosage.

After removal of the medium containing the anticancer drugs, each well was rinsed twice with 3 ml Hanks’ balanced salt solution, overlaid with 4 ml PCM-2 medium (Primaster^®^ serum-free medium), and incubated for a further 7 days. At the end of the incubation, a neutral red solution was added to each well at a final concentration of 50 μg/ml, and colonies in the collagen gel droplets were stained for 2 h. Each collagen droplet was fixed with 10 % neutral buffered formalin, washed in water, air dried, and quantified by optical density image analysis using the Primage System (Solution Systems, Tokyo, Japan). Samples with an optical density >3.0 in the control wells were regarded as evaluable samples. In vitro sensitivity was expressed as the T/C ratio, where T is the optical density of the treated samples and C is the optical density of the controls; a T/C ratio <60 % was regarded as chemosensitive in vitro. The cutoff value at 60 % was used in the current clinical trial because the percentage of patients determined as chemosensitive had been 29.6, 28.6, and 47.3 % for 5-FU, CPT-11, and TXT, respectively, among 30 samples tested by the same investigators in a preparatory pilot study. These percentages were relatively close to the response rates of each drug in the clinical setting.

### Study design, patient allocation, and treatments

**Fig. 1:**
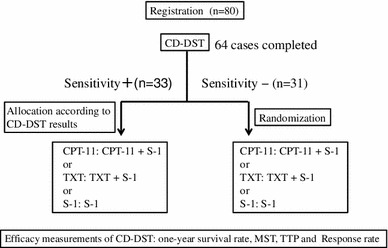
Allocation of chemotherapy on the basis of collagen gel droplet embedded culture-drug sensitivity test (CD-DST) results

**Fig. 2:**
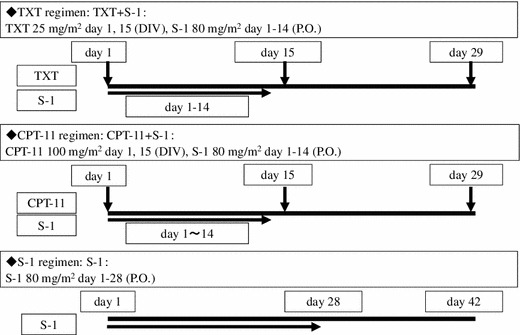
Chemotherapy schedule

We hypothesized that therapy with anticancer drugs to which patients were deemed sensitive would be more effective than therapy with anticancer drugs that were blindly selected. Based on this hypothesis, we opted for a nonrandomized method where patients were allocated to personalized anticancer drugs predetermined by CD-DST. Patients were allocated to one of the following three treatment regimens: TXT/S-1 (TXT), CPT-11/S-1 (CPT), or S-1 (S-1; Fig. 1). Briefly, when CD-DST results showed sensitivity to all three anticancer drugs, patients were allocated to the regimen with the drug predicted to be most effective, that is, the drug with the lowest T/C ratio (sensitive group). When CD-DST results showed sensitivity to either TXT or CPT-11, or only to S-1, patients were allocated to the regimen with the corresponding drug (sensitive group). When cancer cells were not sensitive to any of the drugs, patients were randomly allocated (resistant group). Figure 2 shows details of the TXT, CPT-11, and S-1 regimens.

Treatment was discontinued in the event of serious adverse events, disease progression, or patient refusal, or when the physician in charge decided that the treatment should be discontinued. Further lines of treatment were to be given at the discretion of the physicians.

In the aforementioned pilot study with a cutoff value of T/C ratio at 60 %, 8 of 30 samples (26.4 %) were deemed chemoresistant to all three drugs. The difference in 1-year survival rate between these patients and 16 patients who were determined as chemosensitive was 25 % (the chemosensitivity test failed in the other 6 patients). To detect a similar difference in 1-year survival rate at *α* = 0.05 and *β* = 0.2, the sample size of the study was calculated to be 144 patients, of whom 38 patients were expected to be rated as chemoresistant.

### Patient evaluation

The primary endpoint was 1-year survival rate. The secondary endpoints were time to progression (TTP), median survival time (MST), and response rate. The response was evaluated in accordance with the Response Evaluation Criteria in Solid Tumors (RECIST). Acute toxicity was graded according to National Cancer Institute Common Toxicity ver. 3.0.

### Statistical analysis

Survival curves were calculated according to the Kaplan–Meier method. A generalized Wilcoxon test was used to determine significant differences between curves. The chi-squared test and Student’s *t* test were used to determine differences between groups. *P* < 0.05 was considered statistically significant.

## Results

### Patient accrual

Results of pivotal phase III trials emerged while our trial was ongoing. A regimen of CDDP plus S-1 have shown significant improvement in overall survival over S-1 monotherapy in a randomized comparison for advanced gastric cancer (SPIRITS trial) [6]. More recently, a regimen consisting of capecitabine, CDDP, and trastuzumab whose efficacy was proven in the ToGA trial became a standard chemotherapy for HER2-positive advanced gastric cancer [7]. Because results of the SPIRITS trial were promptly reflected in the Japanese Gastric Cancer Treatment Guidelines 2010 and a combination of S-1 and CDDP became the standard of care, the Shiga Clinical Study Group for Chemosensitivity Tests for Gastrointestinal Cancer decided that offering other regimens as a first-line therapy constituted an infringement of ethics. The final decision was that the trial should evaluate 80 patients who had already completed the 1-year follow-up at the time of decision (total number of patients recruited at the time was 129). Of these 80 patients, CD-DST results were successfully obtained in 64 patients (80 %). Failure to obtain results in the remaining 16 patients (20 %) was mainly the result of contamination from bacterial infection. Thus, 64 patients with CD-DST results were further assessed (Table 1).

**Table 1:** Patient characteristics

	Sensitive group	Resistant group	*p*
Sex			
Male	26	25	
Female	7	6	0.649
Age	65.3 ± 9.9	65.7 ± 10.2	0.468
Histology			
pap	0	0	
tubl	3	2	
tub2	9	9	
porl	5	9	
por2	8	7	
sig	5	3	
muc	1	1	
endocrine	2	0	0.891
Cycle of chemotherapy	6.7 ± 5.3	6.0 ± 2.8	0.379
Primary tumor			
Palliatively resected (metastatic lesions remain)	28	24	
Unresected	4	5	
Recurrent	1	2	0.422
Chemotherapy regimen			
TXT + S-1	20	11	
CPT-11 + S-1	10	10	
S-l	3	10	0.017

*pap* Papillary adenocarcinoma, *tub1* well-differentiated tubular adenocarcinoma, *tub2* moderately differentiated tubular adenocarcinoma, *por1* poorly differentiated adenocarcinoma, solid type, *por2* poorly differentiated adenocarcinoma, non-solid type, *sig* signet-ring cell carcinoma, *muc* mucinous adenocarcinoma, *endocrine* endocrine carcinoma, *palliatively resected* primary tumor was resected but metastatic lesions remained, *unresected* not possible to resect the primary tumor; chemosensitivity test performed with biopsy specimens, *recurrent* metastatic lesions were resected; metastatic lesions were resected and subjected to the chemosensitivity test

### Patient characteristics

The sensitive group comprised 33 patients and the resistant group comprised 31 patients. Characteristics, allocated regimens, and cycles of chemotherapy in both groups are shown in Table 1. Because the chemotherapeutic regimen in the sensitive group was allocated on the basis of sensitivity results whereas the allocation in the resistant group was random, the number of patients undergoing S-1 monotherapy turned out to be significantly smaller in the sensitive group than in the resistant group.

### Efficacy

**Fig. 3:**
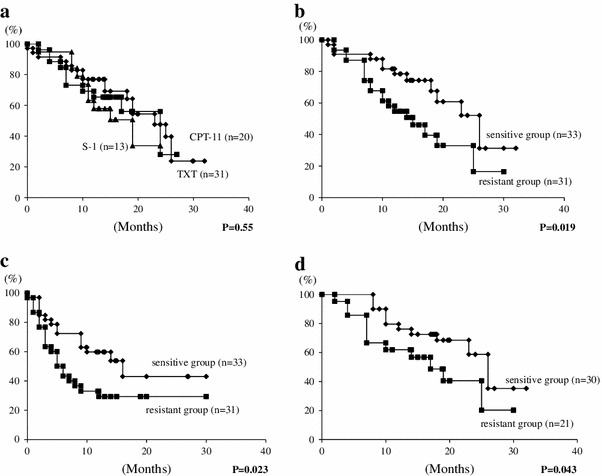
Survival curve on the basis of chemotherapy regimen. **a** No significant difference in survival was noted between the three regimens. Survival curve on the basis of CD-DST sensitivity. **b** The survival rate was significantly higher in the sensitive group than in the resistant group. Time to progression on the basis of CD-DST sensitivity. **c** TTP was significantly longer in the sensitive group than in the resistant group. Survival curve on the basis of CD-DST sensitivity except for S-1. **d** The sensitive group showed significantly better survival than the resistant group

No significant difference in survival was noted among the three regimens (Fig. 3a). The 1-year survival rate was significantly higher in the sensitive group (78.5 %; 95 % CI, 67.2–94.7 %) than in the resistant group (54.7 %; 95 % CI, 38.7–74.3 %; *P* = 0.019; Fig. 3b). TTP was significantly longer in the sensitive group (59.8 %; 95 % CI, 48.2–81.7 %) than in the resistant group (30.0 %; 95 % CI, 13.6–46.4 %; *P* = 0.023; Fig. 3c). MST was also significantly longer in the sensitive group (15.5 months; 95 % CI, 12.8–18.2) than in the resistant group (12.5 months; 95 % CI, 10.2–14.9; *P* = 0.038).

Because the number of patients undergoing the S-1 regimen was significantly lower in the sensitive group than in the resistant group, we reevaluated the survival curves after excluding patients who were treated by S-1 alone from both groups. In this analysis, the sensitive group showed significantly better survival (79.0 %; 95 % CI, 68.5–96.4 %) than the resistant group (62.0 %; 95 % CI, 44.1–85.9 %; Fig. 3d; *P* = 0.043).

The confirmed disease response rate was 45.4 % in the sensitive group (95 % CI, 20.3–73.1 %) and 32.3 % in the resistant group (95 % CI, 7.2–67.7 %). There were no statistical differences (*P* = 0.450; Table 2).

**Table 2:** Response rate

Response	Sensitive group (*n* = 33) (%)		Resistant group (*n* = 31) (%)	
Complete response	1	3.0	0	0.0
Partial response	14	42.4	10	32.3
Stable disease	11	33.3	11	35.5
Progressive disease	7	21.2	10	32.3
Disease control rate	26	78.8	21	67.7
95 % CI		58.2–92.8		44.1–86.6
Overall response rate	15	45.4	10	32.3
95 % CI		20.3–73.1		7.2–67.7

### Toxicity

Adverse events are summarized in Table 3. The incidence of adverse events tended to be lower in patients who underwent the S-1 regimen, but no statistically significant difference was found among the three arms. The incidence of adverse events with toxicity grade 3 or more in the patients who underwent the TXT regimen was comparable to that in patients who underwent the CPT-11 regimen. Temporary discontinuation of chemotherapy was necessary in some cases, but there were no treatment-related deaths.

**Table 3:** Adverse effects of chemotherapy

	TXT regimen		CPT regimen		S-1 regimen	
	All grades (%)	Grade 3 or 4 (%)	All grades (%)	Grade 3 or 4 (%)	All grades (%)	Grade 3 or 4 (%)
Leucopenia	27.0	3.8	36.8	10.5	8.3	0.0
Anemia	52.0	11.1	35.0	0.0	27.3	0.0
Thrombocytopenia	4.2	0.0	11.8	0.0	0.0	0.0
Liver dysfunction	25.0	4.2	5.9	0.0	9.1	0.0
Alopecia	33.3	0.0	38.9	0.0	9.1	0.0
Nausea	33.3	16.7	72.2	5.5	9.1	0.0
Vomiting	12.5	0.0	31.6	5.3	0.0	0.0
Diarrhea	34.5	16.7	44.4	0.0	33.3	0.0
Anorexia	40.0	4.0	63.2	10.5	25.0	8.3
Oral ulcer	20.0	0.0	29.4	0.0	9.1	0.0
Fatigue	30.8	0.0	72.2	16.7	40.0	26.7

## Discussion

The efficacy of molecularly targeted drugs is often predicted by analyzing protein or gene expression. For example, HER2 expression is a predictor of the efficacy of trastuzumab [7], whereas epidermal growth factor receptor (EGFR) expression and the mutation status of the K-Ras gene have been shown to be predictors of cetuximab and panitumumab efficacy [8, 9]. In contrast, pursuit of biomarkers that reflect chemosensitivity of cytotoxic agents had been more problematic, and the in vitro chemosensitivity test remains a practical option in prediction of response to chemotherapy. CD-DST is a chemosensitivity test wherein isolated tumor cells are embedded in collagen droplets. This three-dimensional culture system provides CD-DST with the following advantages over conventional methods: small specimens can be used; the effect of anticancer drugs at physiological concentrations can be assessed; and the masking effect caused by fibroblast contaminations in culture can be eliminated (with the aid of an image analysis system). Consequently, the system provides results that reflect only the anticancer drug effect on cancer cells [4, 5]. Although CD-DST was used to assess sensitivity to S-1, TXT, and CPT-11 in the present study, any anticancer agents can be tested by this method. It may be useful to predict the efficiency of CDDP/5-FU combination chemotherapy, a regimen commonly used worldwide.

The efficacy of CD-DST in cancer treatment has previously been demonstrated [10–13]. CD-DST also proved useful in chemotherapy for residual or recurrent non-small cell lung cancer [14] and for predicting the effect of preoperative chemotherapy for tumor size reduction in patients with advanced or recurrent breast cancer [15]. When outcomes of adjuvant chemotherapy with S-1 were examined in gastric cancer patients, 3-year survival rates and relapse-free survival rates were significantly higher in those with high chemosensitivity [16]. However, to the best of our knowledge, the efficacy of CD-DST in the treatment of advanced gastric cancer has not been studied previously.

We found that 1-year survival rates, TTP, and MST were significantly improved when treated with drugs predicted by CD-DST to be sensitive. Because of the small number of patients, our results may not be sufficient to claim the efficacy of CD-DST in gastric cancer treatment, but they do indicate the potential for CD-DST in selecting anticancer drugs for use in personalized medicine. One drawback is a possibility that the inferior survival time of the resistant group merely reflects more aggressive biology, theoretically associated with inclusion of greater proportion of cancer stem cells or cancer cells that have undergone epithelial to mesenchymal transition. A larger trial with more sophisticated design is needed to overcome this argument.

S-1 is the most frequently used type of fluoropyrimidines for treating gastric cancer patients in Japan. TXT and CPT-11 are also commonly used. When we embarked on this study, TXT/S-1 and CPT-11/S-1 had been regarded as promising candidates for standard first-line chemotherapy for gastric cancer and had actually been under evaluation in phase III trials with S-1 monotherapy as a control. It was only after a series of phase III trials that S-1/CDDP became the standard first-line treatment in Japan. In non-Asian countries, combinations such as infusional 5-FU/CDDP, capecitabine/CDDP, and capecitabine/oxaliplatin have been frequently prescribed for treating advanced gastric cancer. S-1/CDDP was also approved in 30 European countries after the favorable safety profile was revealed in the FLAGS trial [3]. Thus, a combination of oral or infusional fluoropyrimidine with a platinum agent can be considered as the current standard of care for gastric cancer worldwide. In the current study, no significant difference in survival was noted among the three regimens, and no comparison was made between these and S-1/CDDP. However, the main purpose of this study was to examine the relevance of CD-DST in the treatment of advance gastric cancer and not to make comparisons in efficacy between various treatment regimens. Our results suggested that a personalized therapy guided by adequate chemosensitivity testing could lead to superior outcome when compared with the standard treatment. To robustly clarify this issue, a larger trial as proposed by Schrag et al. [17] might be necessary. In that trial, patients in the exploratory arm receive one of several treatments including S-1/CDDP based on the chemosensitivity test while all patients in the control group receive S-1/CDDP.

Despite the introduction of several new promising anticancer drugs, none appears to produce satisfactory outcomes in patients. S-1 was shown to significantly improve the survival rate of gastric cancer patients when used as adjuvant chemotherapy after gastrectomy [18]. However, S-1 as a monotherapy was merely noninferior to the conventional infusional 5-FU in the treatment of advanced gastric cancer. Meanwhile, phase III studies comparing single agent (S-1 alone) and its combination with newly approved drugs (TXT plus S-1 or CPT-11 plus S-1) eventually showed no difference in survival rates [19, 20]. In the present study, the 1-year survival rate in both combination chemotherapy regimens was higher in the sensitive group in terms of CD-DST than in the resistant group. If CD-DST had been employed in the aforementioned phase III studies, a significant survival advantage might have been observed in the patients who were shown to be chemosensitive for TXT or CPT-11. The notion of incorporating chemosensitivity testing with a relevant phase III trial as proposed by Wieand [21] may thus be another means of validating the concept of the chemosensitivity assay. According to his concept, investigating CD-DST results in both responder and nonresponder subgroups in future phase III trials of anticancer drugs will confirm the clinical significance of CD-DST in chemotherapy.

To conclude, a superior 1-year survival rate was observed among chemosensitive patients who received CD-DST-guided treatment when compared with chemoresistant patients for whom the treatment was randomly allocated. Thus, CD-DST might be helpful for selecting appropriate anticancer drugs in the treatment of advanced gastric cancer.
